# Vaccination-hesitancy and global warming: distinct social challenges with similar behavioural solutions

**DOI:** 10.1098/rsos.211515

**Published:** 2022-06-15

**Authors:** Ilan Fischer, Daniel I. Rubenstein, Simon A. Levin

**Affiliations:** ^1^ Department of Psychology, University of Haifa, Haifa, Israel; ^2^ Department of Ecology and Evolutionary Biology, Princeton University, Princeton, NJ, USA

**Keywords:** vaccination hesitancy, global warming, behaviour, SERS‌

## Abstract

Although the COVID-19 vaccine has dramatically changed the fight against the pandemic, many exhibit vaccination-hesitancy. At the same time, continued human-induced emissions of greenhouse gases pose an alarming threat to humanity. Based on the theory of Subjective Expected Relative Similarity (SERS) and a recent international study that drastically modified COVID-19 health-related attitudes, we explain why a similar approach and a corresponding public policy are expected to help resolve both behavioural issues: reduce vaccination hesitancy and motivate climate actions.

## 1. Introduction

The remarkable and rapid development of COVID-19 vaccines has provided incredibly effective tools in fighting the pandemic [1]. Nevertheless, large parts of the population exhibit hesitancy to take the vaccine and oppose its enforced administration, even in countries that provide the vaccine at little or no cost and encourage vaccination [2]. In a parallel manner, global warming, driven by human-induced emissions of greenhouse gases, threatens food security, water availability and habitability of entire regions, and is perhaps the greatest threat to global health in the twenty-first century [3]. Nonetheless, many individuals fail to acknowledge the severity of the situation, deny its existence and impact, discount the societal benefits of preventing it, and avoid taking action. Attenuating the influence of global warming requires making radical changes in international and governmental policies, diverting corporate and industrial norms, and inducing profound changes in individuals' conduct. Elinor Ostrom wrote that ‘To solve climate change in the long run, the day-to-day activities of individuals, families, firms, communities and governments at multiple levels—particularly those in the more developed world—will need to change substantially’ [4]. Inducing changes within all levels necessitates gigantic investments, powerful legislation and effective governance. Moreover, inducing collective changes requires developing the underlying and implicit cooperative *attitudes* among the wider population and the main actors, including corporates and political institutions.

As shown in a recent international study, which successfully improved COVID-related health attitudes [5], inducing change in individuals' motivation is an achievable task. Nevertheless, it requires, not only technical solutions but also a profound understanding of the strategic interactions and decisions made by the individual.

**Figure 1.**
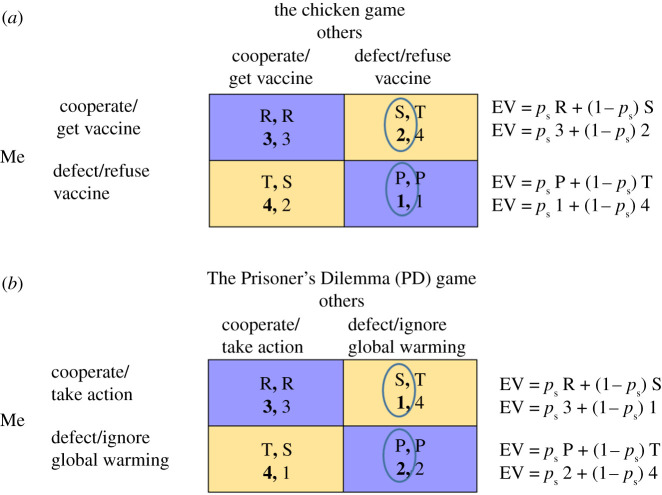
Vaccination hesitancy and global warming behavioural choices modelled as two Similarity Sensitive Games—the Chicken and the Prisoner's Dilemma game, played by an individual who considers the wider public (others) as the opponent. The payoffs in each cell (left for the row player—Me, right for the column player—Others) are presented by their classical notations: Temptation (T), Reward (R), Punishment (P) and Sucker (S). These payoffs are described by the following inequalities for the Chicken game: T > R > S > P, and T > R > P > S, for the PD game. The payoffs are also denoted by their rank orders (1,2,3,4) in the bottom line of each cell. When others are considered to be completely similar (*p*_s_ = 1, purple cells) the individual (Me) is expected to prefer the payoff in the upper left cell over the payoff in the lower right cell. When others are regarded as completely dissimilar (*p*_s_ = 0, yellow cells) the individual (Me) is expected to prefer the payoff in the lower left cell over the payoff in the upper right cell. When 0 < *p*_s_ < 1 the players' preferred choice is determined by comparing SERS's Expected Values (EV) of both alternatives [13,14]. The exact Expected payoffs, EVs, for the row player, as derived from SERS, are indicated on the right side of each game matrix (once by incorporating the variables, and once by illustrating the differences between the games by assuming the rank orders represent actual payoffs). When both EVs are equal, we may derive the exact similarity threshold of the game, or the switching point between the choice of cooperation and defection, denoted by *p*_s_*. The value of *p*_s_* for both games is given by (T − S)/(T − S + R − P).

To analyze vaccination hesitancy, from the perspectives of individuals, we define the following four scenarios: (1) the individual avoids vaccination, while the entire public or a sufficient number of others take the vaccine (hence generating herd immunity); (2) both the individual and the public take the vaccine; (3) only the individual gets vaccinated, while most others don't; (4) neither the individual nor the public (or a sufficient number of others) take the vaccine. This simple structure can be modelled by a 2 by 2 game, known as the Chicken game [6] (figure 1*a*), a game that may be regarded as a cooperative game. An individual who assumes that an insufficient number of others will be vaccinated should be motivated to protect himself or herself by taking the vaccine, as both the risks of not being protected and the advantages of being protected become more crucial for one's own health. On the other hand, an individual who expects a sufficient number of others will get vaccinated, hence generating herd immunity, may be motivated to avoid getting the vaccine, thereby reducing the prospects of suffering from possible side effects. In terms of classical game-theory, both outcomes are Nash equilibria [7], hence providing an outcome that no player is motivated to leave unilaterally (i.e. change his or her choice while assuming the other party does not change his or her choice). Still, taking the vaccine allows players to *minimize their maximal losses* (S > P; where losses, either S or P, refer to the smallest payoffs one may obtain under each of the alternatives, figure 1*a*) and provides a better outcome when one expects others to avoid the vaccine (again S > P, figure 1*a*). In reality, some individuals choose to cooperate, while many others choose to defect, a behaviour suggesting people may be following a different rationale (as explained further below).

Examining the reasoning behind the choice of individuals whether or not to engage in climate actions reveals a different strategic structure or game, that may also be described by four scenarios. (1) the individual avoids taking climate actions, while the entire public, or a sufficient number of others, are seriously engaged in climate actions; (2) both the individual and the public take climate actions; (3) only the individual takes climate actions, while most others don't; (4) neither the individual nor the public (or a sufficient number of others) are involved in climate actions. This simple structure is best described by the Prisoner's Dilemma (PD) game [8] (figure 1*b*). A person or a group that assumes others will not take action is likely to realize that her or his costly contribution will not be enough to make a difference, therefore being motivated to avoid taking part in the necessary actions. Moreover, if one assumes others do take action, one's own contribution may be less critical, again inducing the motivation to free-ride and avoid taking action. In accord with classical game theory, PD players, specifically those who assume they interact in a single-shot game, are destined to avoid climate actions. Avoiding action is a dominant strategy that provides a better outcome under each of the possible choices of others (T > R and P > S, figure 1*b*), thus making other players' behaviours (and therefore also the beliefs about others’ behaviours) irrelevant. Avoiding action allows players to minimize their possible losses (P > S, where losses, either P or S, refer to the smallest payoffs one may obtain under each of the alternatives, figure 1*b*) and to maximize their possible gains (T > R, figure 1*b*). Moreover, mutual avoidance is the only Nash equilibrium of the game, an outcome, or a cell in the matrix (lower right, figure 1*b*), no player is motivated to abandon unilaterally [7]. Importantly, the PD game triggers the scenario termed by Hardin ‘the tragedy of the commons', a situation in which ‘the individual benefits as an individual from his ability to deny the truth even though society as a whole, of which he is part, suffers’ [9]. The tragedy of the commons predicts the collapse of social cohesion and the depletion of public goods and natural resources. However, in contrast to classical game-theoretic reasoning and Hardin's grim perspective [9], when tested in the laboratory (either as a single or a repeated game), many PD players choose to cooperate [8,10–13]. As noted by Ostrom [4] ‘You would not be reading this article if it were not for some of our ancestors learning how to undertake collective action to solve social dilemmas. Successive generations have added to the stock of everyday knowledge about how to instill productive norms of behaviour in their children and to craft rules to support collective action that produces public goods and avoids ‘tragedies of the commons’. What our ancestors and contemporaries have learned about engaging in collective action for mutual defense, child rearing and survival is not, however, understood or explained by the extant theory of collective action’.

Concurring with Ostrom's perception, the theory of Subjective Expected Relative Similarity (SERS) [13], provides an updated and empirically validated rationale that predicts cooperation whenever the subjectively perceived similarity with the opponent exceeds a specific threshold. In accord with SERS, each player, consciously or unconsciously, assigns a probability of *strategic similarity with the opponent*, denoted by *p*_s_, that indicates the prospects of the opponent choosing a similar alternative (and the complementary probability, 1-*p*_s,_ indicating the prospects of the opponent choosing a dissimilar alternative). Then, by calculating Expected Values (EVs) that integrate *these similarity perceptions* with the payoffs of the game, the player weighs both alternatives and chooses the one with the higher EV.

Note that SERS *differs* from the classical game-theoretic perspective, which integrates the beliefs about the opponent by assigning a probability to the prospects of the opponent to cooperate and the complementary probability for the prospects of the opponent to defect. Instead, SERS assigns a probability of strategic similarity, *p*_s_, that indicates the prospects of the opponent choosing a similar alternative. This probability of strategic similarity indicates the prospects of both parties cooperating with each other as well as the prospects of both parties confronting each other. The complementary probability of 1 − *p*_s_ indicates both scenarios where one of the parties cooperates while the other confronts (or defects). While the conceptual change may look small, it generates valid predictions of actual human behaviour. SERS has been empirically validated [13,14], and further developed into a powerful evolutionary computer simulation that outperforms many potent decision rules and learning algorithms [15]. Importantly, SERS is not only applicable to PD and Chicken games (figure 1*a,b*), but also to an extensive category of Similarity Sensitive Games (SSGs) [14,16]; games that comprise 36 out of the 78 games listed in Guyer and Rapoport's taxonomy of 2 by 2 games [17], and is applicable to many if not most ecologically valid conflicts.

Formally applying SERS to the climate action dilemma, as modelled by the PD game, we obtain two Expected Values (EV) for the choices of ‘taking climate actions' (i.e. cooperation) and ‘avoiding climate actions’ (i.e. defection) (EV (Take climate action) = R *p_s_* + S (1 − *p*_s_) and (EV (Avoid climate action) = P *p_s_* + T (1 − *p*_s_), (figure 1*b*)). For example, using the *rank orders* of the payoffs, shown in figure 1*b*, as actual payoffs by defining T = 4, R = 3, P = 2 and S = 1, and assuming the individual considers others to be 60% similar to himself or herself, allows computing two EVs. EV (Take climate actions) = 0.6 × 3 + (1 − 0.6) × 1 = 2.2, and EV (Avoid climate actions) = 0.6 × 2 + (1–0.6) × 4 = 2.8, thus motivating individuals to avoid taking climate actions. Nevertheless, if for the same payoffs the individual assumes others are 90% similar to himself or herself, we obtain EV (Take climate actions) = 0.9 × 3 + (1 − 0.9) × 1 = 2.8, and EV (Avoid climate actions) = 0.9 × 2 + (1 − 0.9) × 4 = 2.2, now inducing the motivation to take climate actions. In general terms, when the perception of similarity with others is sufficiently high, individuals are motivated to take climate actions.

The insertion of *p*_s_ values not only helps with modelling and predicting actual human choices, it also provides a lever that allows motivating cooperative behaviours [13–16]. While the most direct estimate of strategic similarity is derived from observations made along recurrent interactions of the parties, such observations are rare, and may even be completely missing. Instead strategic similarity may be inferred from various cognitive, social and behavioural aspects. Similarity perceptions may emerge from sharing a common history, culture or set of values; from facing identical challenges and hardships; or from having identical aspirations.

The necessity of both sides to rely on such proxies allows players or third parties who aim to increase the extent of cooperation to *provide ample and clear information that indicates the extent of similarity among the interacting parties*. This is not a simple task, modern societies comprise many individuals and groups who do neither feel similar to others, nor to the representatives that shape the public agenda. Such individuals are expected to defect, avoid vaccination and evade taking climate actions. Therefore, the induction of similarity perceptions among various groups and fragments of the society is a critical condition. The more similar others are perceived to oneself, the higher the prospects of cooperation, either by getting vaccinated against COVID-19 or by getting involved in climate actions. This cooperative reaction is a natural and deeply rooted response; and as shown in laboratory experiments [13,14], it does not require participants to formally understand the underlying computational rationale. For this reason, policy makers need to address all parts of the population while making their utmost efforts to reveal and explain why, in spite of the fragmentation of the society, we all share similarity in respect to the choices underlying both contemporary challenges humanity is facing, COVID-19 vaccination and global warming.

Importantly, we cannot wait until the population will gain a natural understanding of the threats, the associated payoffs, and the actual similarity we all share in regard to the contemporary challenges. Naturally evolving perceptions, attitudes and behavioural changes are likely to require long, or even historical, time spans. This is far too slow for dealing with modern-day challenges.

But urgency should not be interpreted as a necessity for strong-arming and enforcement of the appropriate behaviours. Coercion, even mutually agreed upon coercion, as proposed by Hardin [9], is not likely to have the necessary reach, and may motivate reactance and protest rather than cooperation and compliance. Hence, we propose that social scientists, policy makers and public influencers act as catalysts: not forcing actions, but providing the information and the conditions that promote the comprehension of the situation, induce the motivation to act, and provide the means that enable individuals to take actions.

## 2. From theory to practice

To show how such information may be delivered and induce natural attitude changes, we refer to a recently conducted international study that addressed COVID-19 health behaviours and successfully induced meaningful attitude changes [5].

Among several applied interventions, the study involved implicit assessments of initial and final attitudes, provided clear expert advice and delivered a set of simple tasks that enhanced the understanding of the situation. Advice was comprised from objective and up-to-date health instructions provided by professional and non-political experts. Since the extent of advice-taking increases by about 20% following the understanding of the severity of the situation [18], advice-giving was preceded by participants' severity ratings of the pandemic. Special attention was given to the similarity with others. Although the extent of similarity perceived by each individual is impossible to predict, the study raised similarity perceptions by asking participants to think of, and write messages to family members, relatives and people similar to themselves. In this way participants were more likely to surpass the similarity barrier, necessary for making cooperation a rational choice (as defined by SERS). Other tasks asked participants to write short health recommendation thus generating commitment and avoiding a cognitive dissonance by aligning actual behaviours with these self-composed statements. Altogether this study induced a meaningful attitude change, ranging from 36% to 94%, dependent on the specific population and type of practiced intervention, showing that even short, yet thoroughly planned, interventions can induce meaningful changes.

This example shows that changing people's attitudes is possible, but that getting there requires transforming plain information into a meaningful comprehension of the situation and its relevance to the individual. Once achieved, this knowledge translates into new attitudes, which are then expected to drive internally motivated actions. Successfully achieving this transformation should build upon decades of research of human attitudes, cognition, reasoning and decision making.

Our take-home message addressed to policy makers and public influencers is that presenting the information and asking people to conform is not enough. To get compliance, for both COVID-19 vaccination and for the taking of climate actions, individuals need to be able to correctly comprehend the private and public payoffs structure of the interaction they are involved in. Neither broadcasting the information, nor instructing people to follow, is sufficient to induce comprehension and compliance. Most critically, people need to perceive sufficiently high strategic similarity with other involved parties. It is an indisputable fact that many populations around the globe are being neglected or marginalized by central authorities and other socioeconomic stronger populations. These estranged groups will not easily be convinced to perceive central authorities and corporates as being sufficiently similar to themselves. Nevertheless, *all* populations need to be assured that their cooperative efforts, if made, will be answered by the same token. Whenever applicable, public discourse should clearly associate ‘others’ with close family members, next of kin, close friends and individuals who share identical goals, motivations, aspirations and values.

Even if individuals differ in respect to several socio-economic aspects, they still need to be able to perceive similarity when considering common threats, such as COVID-19 and global warming.

Two final comments seem in place. First, while we have addressed the capacity to induce changes in human attitudes and behaviour, such change should go hand in hand with available opportunities to manifest these behaviours. Once people understand the necessity to change various aspect of their life, they need to easily locate available ways to manifest their intentions. A person willing to change the way of commuting should be able to find efficient and practical public transport. A person willing to use green energy should be able to connect to green power networks, and a person willing to reduce meat consumption should find ample, nutritious and tasty supplements. Even a person willing to avoid wasting food should easily find ways to cook and consume all calories, which otherwise would be wasted. Such an initiative has been taken by the US National Research Council's Committee on Food Habits, during WWII [19]. The committee investigated why people eat, what they eat and then practised several methods for changing these habits. In other words, to enable and facilitate behavioural change, people need not only to understand the situation and be motivated to choose cooperative actions but should also find available tools enabling to manifest the changes.

Second, many of the mechanisms associated with individuals' behaviour extend to other levels, including the national and the international arena. As pointed out by Ostrom, ‘To solve climate change, in the long run, the day-to-day activities of individuals, families, firms, communities and governments at multiple levels—particularly those in the more developed world—will need to change substantially’ [4]. Inducing cooperation among governments and corporates, specifically when considering PD (or other SSGs) interactions requires instigating formal mechanisms that assure strategic similarity among key players is kept, to the extent that all parties are likely to narrow their consideration of possible outcomes to the choice between mutual cooperation and mutual defection (i.e. the R and P payoffs in figure 1). The failure to reveal, induce and maintain strategic similarity is likely to jeopardize the prospects of successfully dealing with both contemporary challenges: assuring wide-ranging vaccination against COVID-19 and taking immediate actions to attenuate global warming.

## Data Availability

This article has no additional data.
